# On the Road to Elimination of Rhodesiense Human African Trypanosomiasis: First WHO Meeting of Stakeholders

**DOI:** 10.1371/journal.pntd.0003571

**Published:** 2015-04-02

**Authors:** Peter Holmes

**Affiliations:** 1 Glasgow Centre for International Development, University of Glasgow, Glasgow, Scotland, United Kingdom; 2 WHO Strategic and Technical Advisory Group for Neglected Tropical Diseases, Geneva, Switzerland; IRD/CIRDES, BURKINA FASO

Human African trypanosomiasis (HAT), also known as sleeping sickness, has been a major scourge afflicting populations in Africa in areas where its specific vector, the tsetse fly, thrives. This endemic disease with a very high level of mortality has caused large epidemics in the past and had a major impact on the development of rural populations. Two clinical forms exist, each affecting distinct parts of Africa: a chronic form in West and Central Africa caused by *Trypanosoma brucei gambiense* (>95% of current caseload) and an acute form in East and Southern Africa caused by *T*. *b*. *rhodesiense* (<5% of current caseload).

Confronted with widespread mortality due to HAT, the colonial health systems conducted intensive campaigns during the 1960s that brought HAT under control, with a residual low number of new infections per year. Unfortunately, the ensuing rarity of HAT cases led to a decline in awareness, movement to the bottom of the priority lists, and the neglect of control and surveillance activities. As the disease foci had not been truly eliminated and the ingredients for its transmission were still present, the lack of surveillance allowed HAT to re-emerge and to reach epidemic proportions by the end of the 20th century, when new cases were estimated at around 300,000 per year.

This alarming situation mobilized international efforts to support endemic countries in the revitalization of the fight against the spread of the disease. During the past 15 years, the national sleeping sickness programmes have conducted outstanding work with the support of international groups led by the World Health Organization (WHO) and with the commitment of key pharmaceutical companies and major international donors. Surveillance was reinforced with the deployment of various strategies, most notably mass screening campaigns with treatment of all cases detected. The access to diagnosis and treatment was improved in endemic areas [1,2], and the epidemiological knowledge of the disease was advanced, including detailed and dynamic disease mapping in all affected countries [3]. This has involved strong collaboration and coordination of all these stakeholders and the maintenance of a permanent and open dialogue.

As a result of these synergistic actions, the general situation improved, and the number of new reported cases has fallen steadily (6,228 in 2013) [4]. Based on the observed achievements in the control of the disease, in 2012 the WHO Strategic and Technical Advisory Group for Neglected Tropical Diseases decided to target the elimination of gambiense HAT as a public health problem by 2020 and the reduction to zero incidence of the disease by 2030. The 2020 target was included in the WHO roadmap for elimination and control of neglected tropical diseases [5], and it was defined as the reduction of gambiense HAT incidence to less than one new case per 10,000 people at risk, in at least 90% of foci, as well as fewer than 2,000 cases reported globally [4–6]. In 2013, this elimination target was endorsed by the disease-endemic countries [7–8], the WHO Expert Committee on Control and Surveillance of HAT [6], and the London Declaration on Neglected Tropical Diseases [9]. More recently it was adopted by the 66th World Health Assembly in the resolution WHA66.12 [10]. In March 2014, WHO convened the first WHO meeting of stakeholders for the elimination of gambiense HAT (Geneva, Switzerland), which was attended by participants from national sleeping sickness control programmes, research and development groups, international and nongovernmental organizations, and international donors from the public and private sectors [11]. As a result, the participants issued a declaration for the elimination of gambiense HAT [12].

Regarding rhodesiense HAT, the acute form of the disease affecting East and Southern Africa, there are important specific characteristics that call for an approach that is different from that of gambiense HAT. As opposed to gambiense HAT, in which the reservoir is almost exclusively a human being, rhodesiense HAT is a zoonotic disease with wild and domestic animals as the main reservoirs, which ensures the maintenance of a population of infected tsetse flies that occasionally transmit the disease to humans. This zoonotic nature, and particularly the existence of a wildlife reservoir, makes disease-control objectives more challenging.

On 20–22 October 2014, WHO organized in Geneva, Switzerland, the first stakeholders meeting on rhodesiense HAT. The meeting reviewed the current epidemiological status of the disease and the challenges for advancing towards elimination. Although recently there has been progress in reducing the incidence of rhodesiense HAT, this acute disease with high mortality still holds its epidemic potential. The fight to achieve elimination will need multisectoral (One Health) cooperation and coordination at the national, transboundary, regional, and international levels. The participants indicated that greater attention is to be given to the animal reservoir and the respective roles of livestock and wildlife in different countries and in different ecological situations. They called also for improved and sustained surveillance of infection in humans and animals. The capacity of human resources and infrastructure will need strengthening.

The stakeholders also analysed the current status of important technical assets for rhodesiense HAT control that are needed to assist in achieving the elimination goal. These include faster adoption and better utilization of new tools that are already available, including improved diagnostics and therapies, as well as the development of new diagnostic methods and treatments, more cost-effective vector control tools, and quality assurance systems.

The meeting concluded that the elimination of rhodesiense HAT as a public-health problem (defined as less than one case per 10,000 people at risk) by the year 2020 is achievable, provided that the above-mentioned requirements are addressed.

The mechanisms of collaboration and coordination among stakeholders were established within the WHO network for HAT elimination, and the meeting looked at procedures needed for monitoring and evaluation of the elimination process as well as confirmation of outcomes.

The meeting concluded by issuing a declaration for the elimination of rhodesiense HAT, appealing to the international community at large and to disease-endemic countries in particular for their commitment, political support, and essential resources to achieve the elimination goal. In particular, the appeal calls for the establishment in endemic countries of national coordination bodies that include all sectors concerned with rhodesiense HAT transmission and its impact (i.e., human and animal health, wildlife, and tourism) to bring together and synergize efforts [13]. The declaration of the first stakeholders meeting on rhodesiense HAT elimination can be viewed at http://who.int/trypanosomiasis_african/meeting_declaration_rhodesiense_2014_intro/en/.
